# Variation in Gene Expression Across Infection Status and Elevation in a Hawaiian Honeycreeper

**DOI:** 10.1002/ece3.72078

**Published:** 2025-09-03

**Authors:** Loren Cassin‐Sackett, Katherine M. McClure, Taylor E. Callicrate, Eben H. Paxton, Robert C. Fleischer

**Affiliations:** ^1^ Center for Conservation Genomics, Smithsonian Conservation Biology Institute National Zoological Park Washington DC USA; ^2^ School of Biological Sciences University of Louisiana Lafayette USA; ^3^ U.S. Geological Survey, Pacific Island Ecosystems Research Center Hawai'i National Park Hawai'i USA; ^4^ Species Conservation Toolkit Initiative Brookfield Zoo Brookfield Illinois USA

**Keywords:** adaptation, avian malaria, conservation, emerging infectious disease, rapid evolution

## Abstract

Introduced pathogens exert novel selection on hosts, and although many host species have experienced drastic population declines in the absence of adaptation, some hosts have adapted to highly virulent pathogens. For instance, mosquitoes and *Plasmodium relictum* introduced to the Hawaiian Islands have resulted in extinctions and catastrophic population declines due to avian malaria, particularly in the diverse clade of Hawaiian honeycreepers. However, some species, such as the Hawai'i 'amakihi ( *Chlorodrepanis virens* ), can survive infection. Immunity exists in low‐elevation populations where mosquitoes are abundant, whereas high‐elevation, unexposed populations of 'amakihi display greatly reduced immunity. To explore the basis of adaptation to *P. relictum* in low‐elevation 'amakihi, we sequenced transcriptomes from 24 low‐elevation and 15 high‐elevation 'amakihi. We tested for differential gene expression between (i) infected and uninfected birds and (ii) low‐ and high‐elevation birds. Infected birds showed significant differences in expression across many transcripts with diverse cellular functions involved in different pathways of immune response; eight of the top 13 transcripts blasted to genes previously implicated in immunity to malaria in 'amakihi, and 11 have been identified in other infectious disease systems. Thirteen transcripts showed a trend of higher expression in high‐elevation birds. These transcripts blasted to genes involved in metabolism, blood coagulation, and immune response. Our results provide increasing support for a subset of genes involved in immunity to malaria in 'amakihi and hint at possible antagonistic interactions between response to pathogens and environmental characteristics associated with elevation. Further work clarifying the nature of these interactions could benefit conservation efforts of Hawaiian honeycreepers in upper elevation refugia that are increasingly subject to malaria exposure.

## Graphical abstract

Introduced pathogens exert novel selection on hosts, and some hosts have adapted to highly virulent pathogens. We tested for differential gene expression between (i) avian malaria‐infected and ‐uninfected Hawai'i 'amakihi and (ii) low‐ and high‐elevation 'amakihi. Infected birds showed significant differences in expression across many transcripts with diverse cellular functions involved in different pathways of immune response; eight of the top 13 transcripts blasted to genes previously implicated in immunity to malaria in 'amakihi, and 11 have been identified in other infectious disease systems.

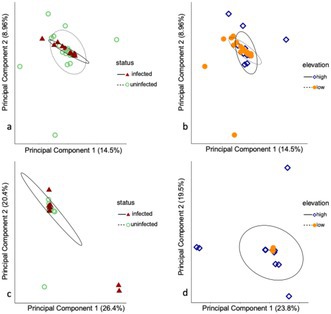

## 1 Introduction

Global anthropogenic change is creating novel selection pressures for species across the planet (Bemmels and Anderson 2019), threatening natural populations with extinction if they do not adapt (DeSaix et al. 2022). However, rapid adaptation to novel selection is common in nature and can occur via nucleotide substitutions in coding genes (Auteri and Knowles 2020), changes in gene expression (Henschen et al. 2023), and structural rearrangements in the genome (Battlay et al. 2024). Adaptation on short time scales is most likely when the selection pressure is strong, provided there is sufficient existing variation in the population.

Strong selection is exerted on naïve organisms by novel pathogens (Cassin‐Sackett et al. 2025; Gignoux‐Wolfsohn et al. 2021; Sackett et al. 2013), in many cases contributing to extinctions (Gilbert et al. 2014; Scheele et al. 2019; Skerratt et al. 2007) and drastic population declines (Cassin‐Sackett et al. 2021; Daszak et al. 2003; Smith et al. 2009). However, adaptation to novel pathogens—although not ubiquitous—has repeatedly been observed in nature (Atkinson et al. 2013; Auteri and Knowles 2020; Epstein et al. 2016; Gignoux‐Wolfsohn et al. 2021; Rocke et al. 2012; Schiebelhut et al. 2018). Much of the early evidence of rapid adaptation to novel pathogens has supported the role of nucleotide changes in protein‐coding genes (Auteri and Knowles 2020; Cassin‐Sackett, Callicrate, and Fleischer 2019; Cassin‐Sackett, Welch, et al. 2019; Epstein et al. 2016; Schiebelhut et al. 2018; Cassin‐Sackett et al. 2025) and microbiome‐conferred resistance (Muletz‐Wolz et al. 2017; Navine et al. 2023; Rebollar et al. 2020; Rosales et al. 2019). Changes in gene expression in immune genes likely also confer immunity to pathogens (Henschen et al. 2023; Paxton et al. 2023), although it is often challenging to demonstrate in nature the role of gene expression in the response of immunologically naïve hosts to new pathogens.

Avian malaria is a globally distributed pathogen that has precipitated population declines and extinctions in naïve species where the pathogen has been introduced (Atkinson and LaPointe 2009). Especially susceptible are the Hawaiian honeycreepers, an adaptive radiation of at least 55 species (Fleischer et al. 1998; Fleischer and McIntosh 2001; James and Olson 1991; Lerner et al. 2011) that diversified from Eurasian rosefinches (Lerner et al. 2011). Though a charismatic example of adaptive radiation, Hawaiian honeycreepers are also a notable example of the consequences of species introduction: As a result of habitat destruction, the introduction of mammalian predators and invasive plants, and non‐native diseases, only 17 species remain today since the arrival of humans to the islands (Atkinson and LaPointe 2009; van Riper III et al. 1986). Since the introduction of the haemosporidian malarial parasite *Plasmodium relictum* in the early 1900s, and the presence of an introduced vector, *Culex quinquefasciatus* (Fonseca et al. 2006), avian malaria has decimated the honeycreepers, likely contributing to 7 extinctions (van Riper III et al. 1986) and population declines in most remaining species (Atkinson and LaPointe 2009; Cassin‐Sackett et al. 2021; Fortini et al. 2015). All but a few Hawaiian honeycreeper species have been forced into high‐elevation refugia where disease transmission is low owing to temperature limitations on mosquitoes and *Plasmodium* (LaPointe et al. 2010; Samuel et al. 2015; van Riper III et al. 1986).

**FIGURE 1:**
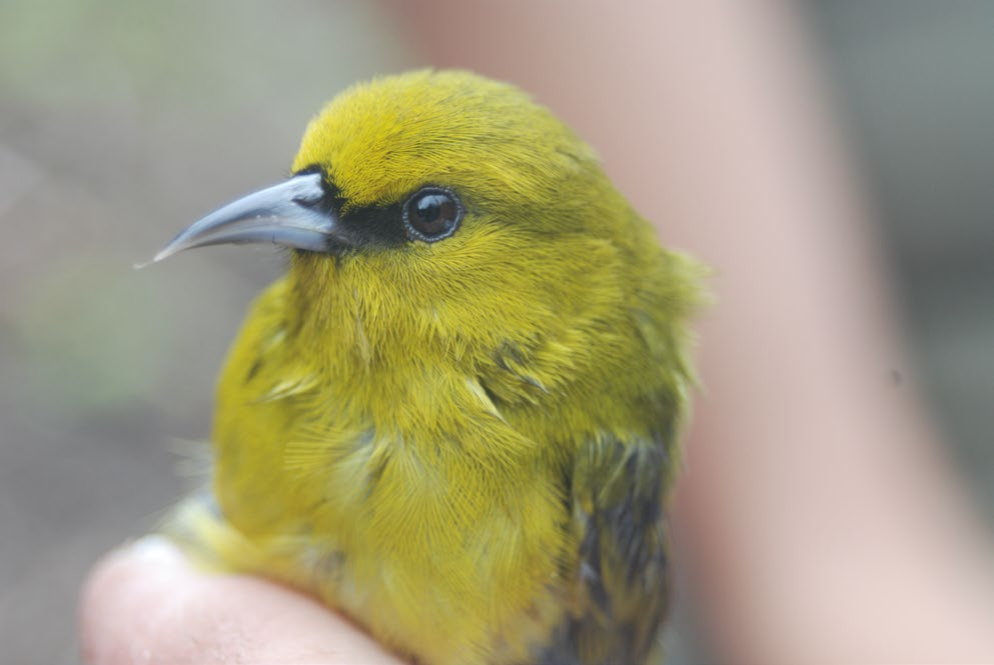
Hawai'i 'amakihi ( *Chlorodrepanis virens* ) from low elevation on the Island of Hawai'i.

Despite the catastrophic consequences of malaria on Hawaiian avifauna, a few species, such as the Hawai'i 'amakihi ( *Chlorodrepanis virens* ; hereafter 'amakihi; Figure 1), have begun to persist in areas with heightened disease transmission: some populations of 'amakihi can survive with low mortality, and infected individuals may experience no decline in fitness (Kilpatrick et al. 2006; but see Kilpatrick 2003). Some 'amakihi populations have persisted at low elevations (Eggert et al. 2008; Foster et al. 2007) despite the high prevalence of *Plasmodium* in both mosquitoes and 'amakihi (Kilpatrick et al. 2006; McClure et al. 2020; Woodworth et al. 2005). The temperature‐limited distribution of mosquito vectors and *P. relictum* leads to elevational gradients in disease transmission, and populations of 'amakihi along that elevational gradient also show different levels of immunity: Challenge experiments have demonstrated higher survivorship of low‐elevation 'amakihi than high‐elevation 'amakihi (Atkinson et al. 2013), and surviving individuals are immune to reinfection (Atkinson et al. 2001). 'Amakihi show higher dispersal within than across elevations (Eggert et al. 2008; Foster et al. 2007; Lindsey et al. 1998), suggesting that low‐elevation 'amakihi populations should display signatures of adaptation in their genomes relative to high‐elevation populations. Indeed, a suite of single‐nucleotide polymorphisms (SNPs) displayed evidence of selection in low‐elevation populations (Cassin‐Sackett, Callicrate, and Fleischer 2019). In experimentally infected 'amakihi, several of these genes and a number of newly identified genes were differentially expressed during infection in survivors relative to fatalities, suggesting adaptation has occurred via the regulation of inflammatory processes (Paxton et al. 2023). It is not currently known which genes are differentially expressed in natural populations in areas of year‐round high transmission (low‐elevation) relative to areas with low or seasonal transmission (high‐elevation) of *P. relictum* on the Island of Hawai'i.

In this study, we implement comparative transcriptomics to examine patterns of gene expression in the endemic Hawai'i 'amakihi. We collected samples from low and high elevation populations on the Island of Hawai'i to compare gene expression in (i) 'amakihi infected with malaria versus those with no detected infections, and in (ii) a population exposed to malaria for > 80 years (low‐elevation) versus a population historically unexposed (high‐elevation) to malaria.

## 2 Methods

### 2.1 Sampling Design, cDNA Library Preparation, and Malaria Testing

**FIGURE 2:**
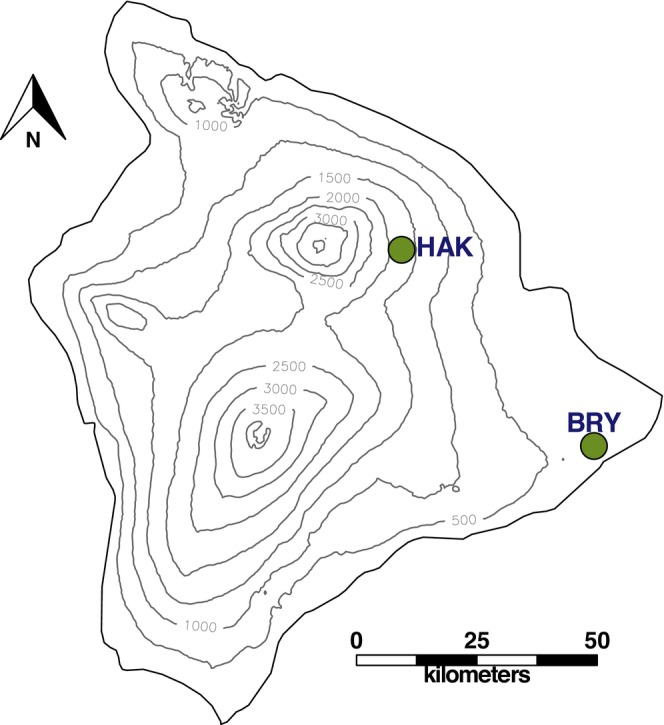
Map of sampling sites on the Island of Hawai'i, with topographic lines (in meters) showing elevation across the island. Sites were classified as low elevation (BRY = Bryson's cinder cone) or high‐elevation (HAK = Hakalau Forest National Wildlife Refuge). Map was generated using R version 4.3.3.

'Amakihi blood was sampled in 2014 from a population on the Island of Hawai'i where mosquitoes and *P. relictum* are present (Bryson's cinder cone (Pu'u Kali'u), “low‐elevation,” 291 masl) and a population where mosquitoes and *P. relictum* have been historically absent (Hakalau Forest National Wildlife Refuge, “high‐elevation,” mean 1666 (range 1524–1890) masl; Figure 2). Sampling followed standard bird mist‐netting and blood collection protocols (Tarr and Fleischer 1993; Woodworth et al. 2005) modified for RNA collection. Specifically, blood was collected from captured birds via brachial venipuncture; one aliquot was placed into Queen's Lysis Buffer (Seutin et al. 1991) for DNA extraction and one aliquot was mixed into RNAlater (Ambion by Life Technologies Inc.) with storage at gradually decreasing temperatures following the manufacturer's suggested protocol. All sampling occurred with required permits (BBL banding permit 21,144 and 23,600) and in concordance with IACUC approval. Total RNA was extracted from blood using the Mouse RiboPure‐Blood Kit (Ambion Inc.) following the modified protocol for blood volumes < 0.25 mL. Subsequently, mRNA was isolated using the MagJET mRNA enrichment in 50 μL volumes, and DNA was digested with Turbo DNase.

cDNA was synthesized in two steps using custom modified reactions for small starting quantities of the SuperScript III First Strand Synthesis system (Invitrogen) followed by the NEBNext mRNA Second Strand Synthesis Module (New England BioLabs). Synthesized double‐stranded cDNA was used as input for a customized dual‐index Nextera‐style library preparation. The transcriptomes of 39 individuals (24 low‐elevation and 15 high‐elevation) were sequenced with Illumina Nextera‐style adapters on two lanes of an Illumina HiSeq 2500 machine at Macrogen.

DNA was extracted using Qiagen's DNeasy Blood and Tissue kit. The presence of *P. relictum* was assessed by amplifying a 160 bp noncoding region of mitochondrial ribosomal RNA of avian haemosporidians using the primers 213F/372R and following the exact PCR reaction mix and cycling conditions described previously (Beadell and Fleischer 2005). PCR bands indicating detection of *P. relictum* were visualized using ethidium bromide‐ or GelRed‐stained agarose gels, and positive and negative controls were included for both the extraction and PCR reactions (McClure et al. 2020).

### 2.2 Sequence Processing and Differential Expression Analysis

We explored multiple trimming and transcriptome assembly parameters (e.g., minimum quality and read length, *de novo* versus reference‐guided assembly) to choose the assembly that resulted in the highest number of concordantly aligned reads across individuals. We used Trimmomatic version 0.39 (Bolger et al. 2014) to trim adapters and quality filter sequences, setting a minimum quality threshold of 14 and retaining only reads that were at least 35 base pairs. Because the majority of quality filtered reads retained their pairs, only paired reads were used for the remainder of the pipeline. We used Trinity version 2.11.0 (Grabherr et al. 2011) to perform *de novo* transcriptome assembly rather than reference‐guided assembly to avoid any bias that could be introduced by aligning reads to the existing 'amakihi genome, which was assembled from a malaria‐infected low‐elevation individual. We evaluated the quality of the assembly in several ways: counting the number of assembled transcripts, generating statistics with Trinity's TrinityStats.pl script, aligning the reads back to the assembly in bowtie2, and generating mapping statistics. Because most of the samples were run in multiple lanes and/or libraries were prepared in duplicate, replicates were processed separately up until this point, when bam files were merged.

We estimated transcript abundance with RSEM, normalizing counts across samples. We conducted a principal component analysis (PCA) on normalized counts across the whole transcriptome and visualized the PCA in R version 4.3.3 (R Core Team 2020). Next, we used the “run_DE_analysis.pl” tool in the Trinity package to test for differential expression between (i) infected birds versus those with no detected infection and (ii) low‐elevation versus high‐elevation birds. The group of uninfected birds contained individuals from both low and high elevations (Appendix A). We used edgeR (Robinson et al. 2010) to visualize heatmaps of expression, and visualized transcripts differentially expressed across elevation and infection status using volcano plots. We conducted a principal component analysis (PCA) on normalized expression across the whole dataset and across subsets of differentially expressed genes using the ade4 package in R (Dray and Dufour 2007), and we used a between‐class analysis (function “bca,” Dolédec and Chessel 1987) with 1000 permutations to test for differences in expression across all principal components between elevation and between infection status. A detailed description of the pipeline and scripts is available on GitHub (https://github.com/CassinSackett/RNA_seq).

The most highly differentially expressed transcripts (see Results) were blasted against Uniprot and NCBI protein databases. Gene function was inferred with a combination of SwissProt and GeneCards summaries and annotations, and gene pathways were inferred with GeneCards/Pathcards. The five most significant (lowest *E* value) matches for each transcript were considered for follow‐up as candidate genes, except in one transcript that had two nearly statistically indistinguishable matches; here, six were considered.

Finally, we examined gene enrichment and depletion in the set of transcripts differentially expressed between infected and uninfected 'amakihi. To do so, we followed the Trinotate pipeline to generate annotations for each transcript, and then used Trinity's “run_DE_analysis.pl” again with the “‐‐examine_GO_enrichment flag.”

To examine possible confounding effects of combining both low‐ and high‐elevation uninfected birds into one group, we performed a second differential expression analysis of low‐elevation birds only, comparing infected (*N* = 14) and uninfected (*N* = 10) birds.

## 3 Results

### 3.1 Samples and Read Statistics

We obtained transcriptomes from 24 low‐elevation and 15 high‐elevation 'amakihi. Fourteen samples, all from low elevation, were positive by PCR for infection with *P. relictum* . No infections were detected in any high‐elevation birds or the remaining 10 low‐elevation birds (hereafter these samples are referred to as “uninfected;” Appendix A). One low‐elevation bird also had a lesion on its foot consistent with infection from avian pox (Eibner‐Gebhardt et al. 2025), another mosquito‐transmitted pathogen introduced to the Hawaiian Islands (Jarvi et al. 2008; Samuel et al. 2018).

The mean number of read pairs per sample was 1.92 million (3.85 million total) and was similar for low‐elevation and high‐elevation samples (2.02 million vs. 1.77 million, unpaired *t*‐test *p* = 0.79). After quality filtering in Trimmomatic, a mean of 1.03 million read pairs per sample remained; the number of remaining read pairs did not differ between low‐elevation and high‐elevation samples (unpaired *t*‐test *p* = 0.38). There was a mean concordant alignment rate of 59.8% and a mean overall alignment rate of 86.3% to the *de novo* assembly.

### 3.2 Differential Expression

**FIGURE 3:**
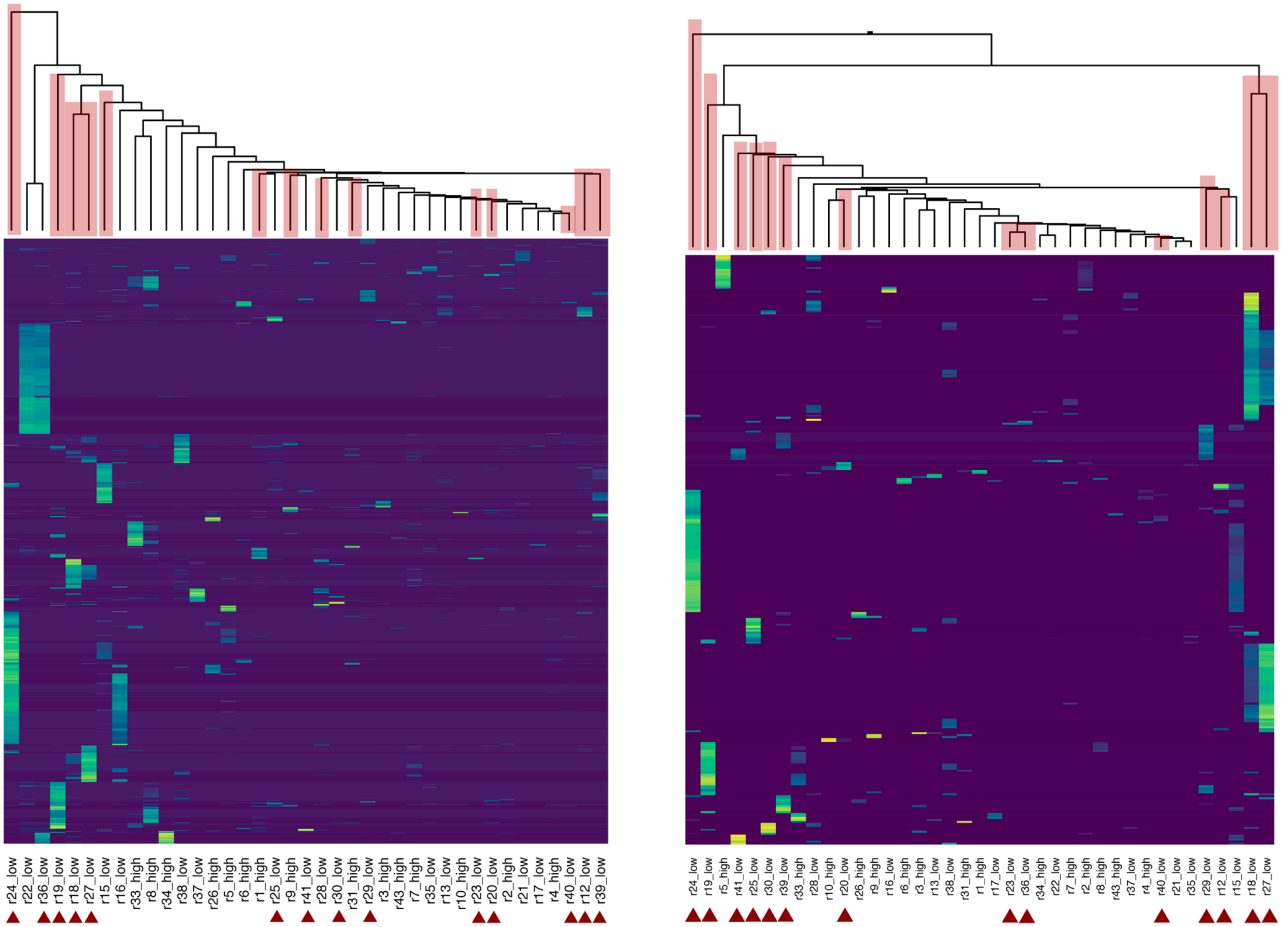
Heatmaps of normalized expression in all 39 birds (columns) across transcripts (rows). Individual birds are in columns, with dendrograms showing clustering at the top and bird ID along the bottom. Infected birds are denoted by dark red triangles below the sample names and by translucent dark red shading over the dendrogram tips. Dark purple shade indicates no expression at that transcript, and brighter colors indicate higher expression. Left: 1751 transcripts included in the EdgeR analysis, showing little clustering by elevation or infection status; Right: The top 299 transcripts significantly differentially expressed (FDR < 0.05) between infected and uninfected 'amakihi; most uninfected birds cluster together in the center of the dendrogram.

**FIGURE 4:**
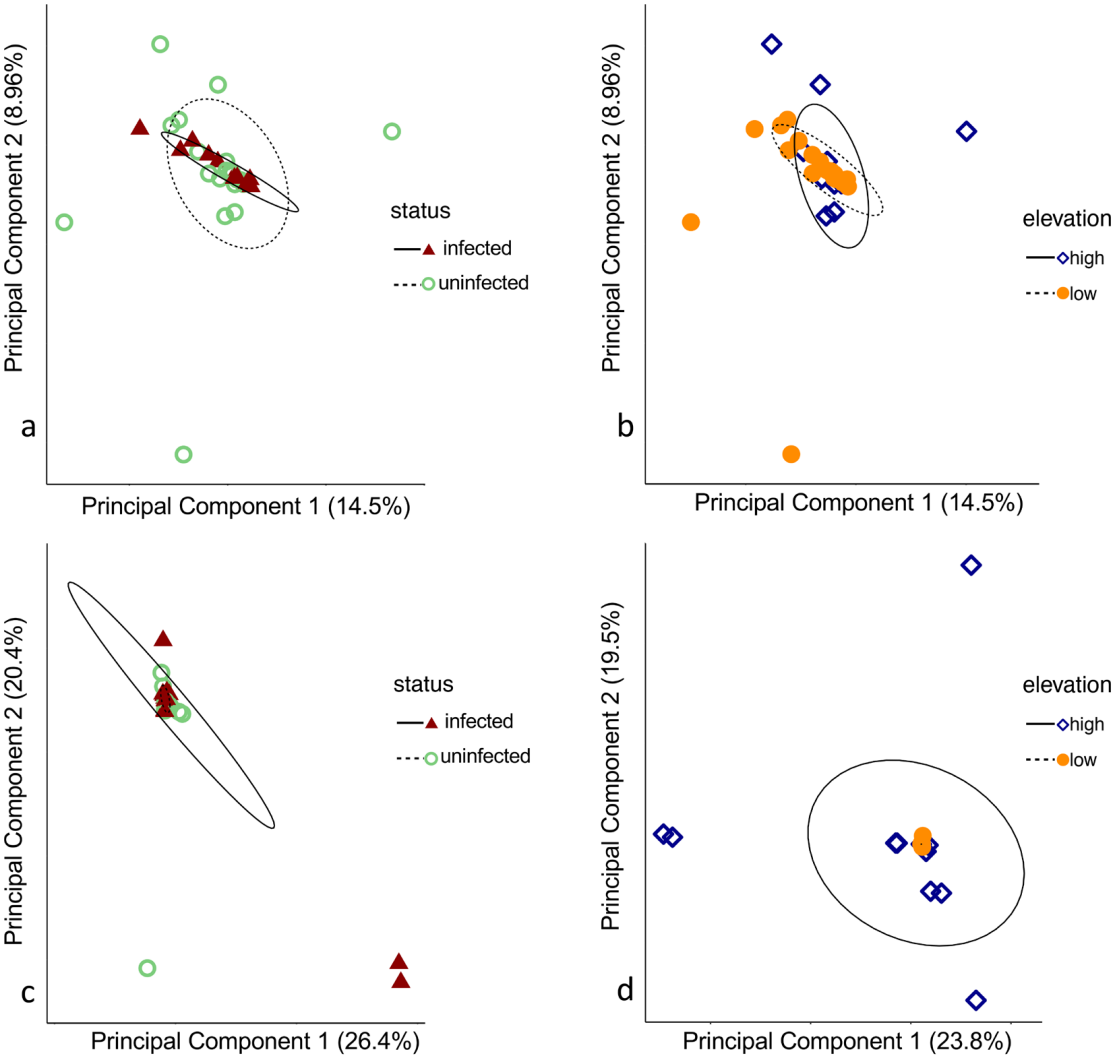
Principal Component Analyses of normalized read counts; infected birds are represented by dark red closed triangles and uninfected birds by light green open circles, and low‐elevation birds are represented by light orange filled circles and high‐elevation birds by dark blue open diamonds. Expression profiles (a) across the transcriptome, with points coded by infection status, (b) across the transcriptome, with points coded by sampling elevation, (c) across the 299 transcripts that show differential expression (FDR < 0.05) between infected and uninfected birds, and (d) and at the 13 transcripts that show a nonsignificant trend of differential expression (unadjusted *p* < 0.01) between elevations. In plots (a) and (b), two individuals were removed from the visualization but not the analysis. In plots (c) and (d), one individual was removed from the visualization but not the analysis. Confidence ellipses were calculated including all individuals.

**FIGURE 5:**
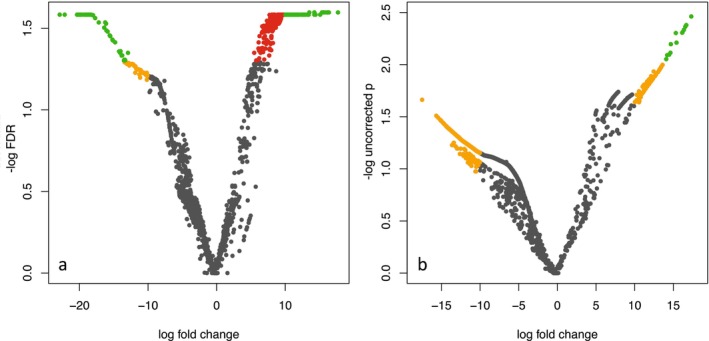
Volcano plots showing log fold change (x‐axis) and −log(*p* value) (y‐axis) of 1751 transcripts in the edgeR analysis. Transcripts colored in red are significantly differentially expressed (*p* < 0.01), in orange have log fold change > 10, and in green have both log(fold change) > 10 and *p* < 0.01. (a) Transcripts differentially expressed between infected and uninfected birds. Green transcripts have FDR < 0.026 and unadjusted *p* < 2 × 10e^−4^. (b) Slight nonsignificant differences in expression between low‐elevation and high‐elevation birds; green transcripts have unadjusted *p* < 0.01 but FDR = 0.16. Note difference in y‐axis between panels: Left panel is −log(FDR‐corrected *p*) and right panel is ‐log(unadjusted *p*).

Most transcripts were characterized by expression in only a small number of birds (Figure 3), likely due to the low mean number of reads per sample and high heterogeneity in coverage across individuals and transcripts. Across the whole transcriptome (*N* = 75,324 Trinity transcripts), normalized gene expression profiles were broadly similar in all birds, without respect to infection status or sampling location (Figure 4a). However, malaria‐infected birds showed significant differential expression (FDR < 0.05) of 299 transcripts relative to uninfected birds (Figure 4b); differences in 13 of these transcripts were highly significant (FDR < 0.026; unadjusted *p* < 0.001). Among the 299 differentially expressed transcripts, 41 showed higher levels of normalized expression in uninfected birds, while 258 showed higher expression in infected birds (Figure 5).

In contrast, there were no significantly (FDR < 0.05) differentially expressed genes between low‐ and high‐elevation samples. However, there were 13 transcripts that showed a nonsignificant trend of differential expression between groups (FDR = 0.1604 and unadjusted *p* < 0.01; green points in Figure 5).

In our comparison of gene expression between infected and uninfected birds from low elevation only, the low sample size (*N* = 14 vs. *N* = 10) combined with heterogeneity in sequencing depth among individuals and transcripts resulted in the majority of transcripts being expressed in very few samples. For instance, all but one transcript was expressed in < 8 samples, and the mean number of samples per transcript was 2.1 birds. Although there were 81 significantly differentially expressed transcripts (FDR < 0.05), only four of these transcripts were represented by > 1 bird per group. Therefore, because the observed differential expression may not be representative of the gene regulatory response of infected birds, we elected not to follow up on these transcripts, focusing instead on our primary analysis of infected versus uninfected birds from all elevations.

### 3.3 Gene Identity and Ontology

Among the 299 transcripts differentially expressed between infected and uninfected birds, none showed depletion of gene ontology categories. The 33 significantly enriched (FDR < 0.05) gene ontology categories were all in genes that were upregulated in uninfected birds; among these, the top three most enriched biological processes were all related to positive regulation of apoptosis and programmed cell death. Other enriched biological processes included several related to the negative regulation of transcription, negative regulation of biosynthetic processes, and negative regulation of metabolic processes. Collectively, this shows the enrichment of processes interrupting the cell cycle and causing cell death in genes upregulated in uninfected birds.

The cellular function of the 13 transcripts that were most significantly differentially expressed between infected and uninfected birds was diverse. Yet almost all have previously identified roles in immune response in various host and pathogen systems (Table 1), many in a regulatory role. Eight of the 13 transcripts with the highest degree of differential expression between infected and uninfected birds blasted to genes that have previously been implicated in survivorship from malaria in Hawai'i 'amakihi (Cassin‐Sackett, Callicrate, and Fleischer 2019; Paxton et al. 2023); these genes were either differentially expressed across experimental groups and/or time points in an experimental infection of 'amakihi with *P. relictum* (Paxton et al. 2023) or inferred to be under selection in a comparison of high‐ and low‐elevation ʻamakihi (Cassin‐Sackett, Callicrate, and Fleischer 2019). Of the 13 most significantly differentially expressed transcripts, three others have been identified in infections of mammals with other apicomplexan parasites (including *Plasmodium berghei*) and bacterial and viral infections (Table 1).

**TABLE 1:** Blast search results for the 13 most significant differentially expressed transcripts between infected and uninfected 'amakihi.

Transcript	Blast hit	Function	In literature findings
TRINITY_DN21866_c0_g1_i1	1. RNA‐binding motif, single‐stranded‐interacting protein 3 (RBMS3) (*E* = 2e‐10)	1. DNA replication, gene transcription, cell cycle progression, and apoptosis	1. May regulate response to malaria infection in mice via interferon pathways (Laroque 2016); inhibits cancer/cell proliferation and induces apoptosis (Chen et al. 2012)
	2. Beta, beta‐ carotene 9′,10′‐oxygenase (BCO2) (*E* = 2e‐10)	2. Oxidizes carotenoids such as beta‐carotene during the biosynthesis of vitamin A	2. Associated with cytokine interleukin‐18 levels (He et al. 2010) and differentially expressed in experimentally infected 'amakihi (Paxton et al. 2023)
	3. Ras‐related protein Rab‐20 (*E* = 3e‐8)	3. Maturation and acidification of phagosomes that engulf pathogens	3. *Plasmodium berghei* induced upregulation of host Rab20 and other Rab genes (Seixas et al. 2012); differentially expressed in surviving 'amakihi at different points of infection (Paxton et al. 2023); associated with survivorship in experimentally infected 'amakihi (Atkinson et al. in review)
TRINITY_DN29122_c0_g1_i1	1. F‐box protein At1g70360 (FB81) (*E* = 0.38)	1. Mediates protein–protein interactions	1. F‐box protein important in parasite growth, cell division, and membrane integrity (Rizvi et al. 2024); multiple F‐box proteins differentially expressed across groups/times in experimentally infected 'amakihi (Paxton et al. 2023)
TRINITY_DN29140_c0_g1_i1	1. Adhesion G‐protein‐coupled receptor F5‐like (ADGRF5) (*E* = 8e‐8)	1. Cell surface receptor signaling pathway	1. Loss of ADGRF5 induces inflammation and immune response (Kubo et al. 2019)
	2. Vomeronasal type‐2 receptor 26‐like (*E* = 2e‐6)	2. Involved in G protein‐coupled receptor signaling pathway	2. Intestinal epithelial Tuft‐2 cells respond to bacterial infection by sensing the bacterial metabolite N‐undecanoylglycine through vomeronasal receptor Vmn2r26 (Xiong et al. 2021)
	3. Uromodulin‐like (*E* = 2e‐5)	3. Inhibitor of calcium crystallization in renal fluids; May serve as a receptor for binding and endocytosis of cytokines (IL‐1, IL‐2) and TNF	3. Differentially expressed in 'amakihi experimentally infected via mosquito vs. blood inoculation (Paxton et al. 2023)
	4. V‐type proton ATPase subunit B (*E* = 3e‐5)	4. Responsible for acidifying and maintaining the pH of intracellular compartments	4. Necessary for parasite survival & digestion of heme (Alder et al. 2023)
	5. Transmembrane protein 260 (*E* = 3e‐4)	5. Integral component of membrane	5. Transmembrane proteins under selection in low‐elevation 'amakihi (Cassin‐Sackett, Callicrate, and Fleischer 2019)
	6. Contactin‐2 (*E* = 4e‐4)	6. Part of the immunoglobulin superfamily of cell adhesion molecules	6. Contactin‐1 downregulated in mouse model infected with malaria (Desruisseaux et al. 2010); contactins 3,5 inferred under selection in low‐elevation 'amakihi (Cassin‐Sackett, Callicrate, and Fleischer 2019); contactin 4 was a candidate malaria resistance locus in humans (Damena et al. 2021)
TRINITY_DN32899_c0_g1_i1	1. Biorientation of chromosomes in cell division protein 1‐like (BOD1) (*E* = 1e‐4)	1. Enables protein phosphatase binding/inhibitor activity	1. BOD1 differentially expressed in fatalities vs. uninfected 'amakihi (Paxton et al. 2023)
	2. Protocadherin gamma‐A6‐like (PCDHGA6) (*E* = 0.002)	2. Potential calcium‐dependent cell‐adhesion protein	2. PCDHGA6 upregulated in infection with Venezuelan Equine Encephalitis Virus (Gupta et al. 2017) & Plasmodium berghei (Desruisseaux et al. 2010); PCDH10 differentially expressed in fatalities before vs. during infection (Paxton et al. 2023); other cadherins are candidate resistance genes to malaria in humans (Mackinnon et al. 2016) & other pathogens in wildlife (Cassin‐Sackett, Tsuchiya, and Dikow 2025)
	3. E3 SUMO protein ligase ZNF451 (*E* = 0.010)	3. Enables SUMO ligase activity and transcription corepressor activity; negative regulation of transforming growth factor beta receptor signaling pathway	3. A different SUMO ligase differentially expressed in infected 'amakihi at different time points (Paxton et al. 2023); ZNF451 the most downregulated gene in Toxoplasma‐infected cells (Elsheikha et al. 2019)
TRINITY_DN3415_c0_g1_i1	1. Cystatin‐C isoform X1 (CST3) (*E* = 0.008)	1. Cysteine protease inhibitor; has an antimicrobial function, inhibiting replication of herpes simplex virus	1. Associated with kidney function and commonly used as a diagnostic of malaria‐induced acute kidney injury (Oluwatuyi et al. 2024)
TRINITY_DN34412_c0_g1_i1	1. Methyltransferase (bacterial) (*E* = 1.2)	1. Transfers methyl groups to other molecules	1. Methyltransferase differentially expressed in infected birds (Paxton et al. 2023)
TRINITY_DN40728_c0_g1_i1	1. Serine/threonine‐protein kinase SMG1 (*E* = 0.021)	1. Plays a central role in nonsense‐mediated decay of mRNAs containing premature stop codons	1. SMG1 likely reduces inflammation (Roberts et al. 2013) but inhibition of SMG1 increases T‐cell and cytokine activity (Vendramin et al. 2024). Other Serine/threonine‐protein kinase PAK 3‐like and 1 differentiated across elevation (Cassin‐Sackett, Callicrate, and Fleischer 2019); PAK3 associated with survivorship from malaria in experimentally infected 'amakihi (Atkinson et al. in review), and SGK3 differentially expressed in groups of experimentally infected 'amakihi (Paxton et al. 2023)
TRINITY_DN49874_c0_g1_i1	1. Putative fanconi anemia group d2 protein (FANCD2) (*E* = 1.1)—mosquito	1. Involved in the repair of DNA double‐strand breaks; May also be involved in B‐cell immunoglobulin isotype switching	1. Fanconi anemia complementation group E differentiated across elevation in 'amakihi (Cassin‐Sackett, Callicrate, and Fleischer 2019; Cassin‐Sackett, Welch, et al. 2019); L and I differentially expressed in survivors reducing parasitemia and in fatalities vs. uninfected 'amakihi (Paxton et al. 2023)
	2. Cytochrome b5 (CYB5) (*E* = 1.5)	2. Enables heme binding activity	2. CYB5 genes downregulated in 'amakihi fatalities (Paxton et al. 2023)
TRINITY_DN38390_c0_g1_i1	1. Cholinesterase (CHLE) (*E* = 0.58)	1. Helps the nervous system function by breaking down acetylcholine	1. Acetylcholinesterase activity higher in mice experimentally infected with Toxoplasma gondii than controls (Tonin et al. 2013); Cholinesterase activity associated with COVID‐19 severity (Nakajima et al. 2021) and whether patients developed septic shock from bacterial infections (Bahloul et al. 2017)
TRINITY_DN42538_c0_g1_i1	1. Bifunctional protein Aas (bacterial) (*E* = 0.19)	1. Involved in lysophospholipid acylation	
	2. Deoxyuridine 5′‐triphosphate nucleotidohydrolase (DUT) (*E* = 0.26)—viral	2. Prevents uracil misincorporation into DNA	2. Horizontal transfer of DUT between host and pathogen in all three domains of life (McClure 2001)
	3. Purine nucleoside phosphorylase (PNPH) (*E* = 0.39)	3. Catalyzes the phosphorolysis of purine nucleosides; important in T‐cell (cell‐mediated) immunity, B‐cell immunity, and antibody responses	3. PNPH upregulated in some strains of Plasmodium‐infected mice (De‐Oliveira et al. 2006) but reduced in others (Carvalho et al. 2009)
TRINITY_DN52437_c0_g1_i1	1. Endoglucanase A (GUNA) (*E* = 3.9) – bacteria	1. Breakdown of cellulose	
	2. Leucine‐rich repeat LGI family member 3 (LGI3) (*E* = 6.7)	2. Regulation of exocytosis	2. A different family member (LGI4) was in a QTL associated with immunity in a mouse infection of Trypanosoma gondii (Souza et al. 2021)
TRINITY_DN58146_c0_g1_i1	1. tRNA modification GTPase MnmE (MNME) (*E* = 3.6) 2. Transposase inhibitor protein from TN5 (*E* = 8.6) 3. Tn5 Transposase (*E* = 9.5)	1. Involved in the synthesis of a tRNA wobble uridine modification 2. Inhibits transposition 3. Catalyzes transposition	2/3. TE expression is induced by viral infections (Hale 2022); TE essential for regulation of interferon genes (Thomson et al. 2009)
TRINITY_DN46635_c0_g1_i1	1. Dynein axonemal heavy chain 1 (DNAH1) (*E* = 1.6)	1. Force‐generating protein of cilia required for sperm flagellum motility	1. DNAH8 associated with survival in experimentally infected 'amakihi (Atkinson et al. in review)

*Note:* The first eight transcripts included genes that were also differentially expressed in experimentally infected 'amakihi (Paxton et al. 2023); the next three transcripts included genes identified in other studies of *Plasmodium* and *Toxoplasma*. E = expect value from Blast search, with lower values indicating more significant matches. Five transcripts did not have blast hits with *E* < 1; here, the best statistically similar hits for each transcript are shown.

In contrast to the diverse functions of transcripts differentially expressed in infected and uninfected 'amakihi, the inferred functions of the thirteen transcripts that showed a trend (FDR = 0.16, unadjusted *p* < 0.01) of differential expression between elevations (Table 2) largely fell into three categories: three transcripts were related to cellular respiration (*COX1*, *COX2*, and *NADH5*), two were involved in platelet formation and adhesion (Acyl‐CoA‐binding protein and von Willebrand factor), and three had roles in pathogen response (Ig‐like domain‐containing protein, Ras‐related protein *Rab‐12*, and Growth Factor Receptor Bound protein 7 (*GRB7*)).

**TABLE 2:** Blast search results for the top 13 transcripts with the largest magnitude of expression difference between low and high elevation birds.

Transcript	Blast hit	Function	In literature findings
TRINITY_DN149_c1_g3_i1	Ig‐like domain‐containing protein (*E* = 1e‐4)^a^	Antigen binding/immunoglobulin mediated immune response^a^	Lower expression in warmer temperatures in yak (Gu 2024); Sema domain, immunoglobulin domain (Ig) semaphorin 3A (SEMA3A) differentiated between low/high elevation 'amakihi (Cassin‐Sackett, Callicrate, and Fleischer 2019)
TRINITY_DN235_c0_g1_i1	Cytochrome c oxidase subunit 2 (*E* = 2e‐34)^b^	Mitochondrial respiratory chain^b^	Changes in COX in bar‐headed geese flying at high altitudes (Scott et al. 2011)
TRINITY_DN25799_c0_g1_i1	2‐nitroimidazole transporter (*E* = 6.5) [bacteria]	Transmembrane transporter activity	Under hypoxia, nitroimidazoles can replace oxygen as an electron acceptor (Koike et al. 2020); hypoxia can influence susceptibility to infections (Dzhalilova and Makarova 2020)
TRINITY_DN28347_c0_g1_i1	Ras‐related protein Rab‐12 (*E* = 2e‐12)^a^	Regulators of intracellular membrane trafficking; endosome to lysosome transport; acts upstream of or within cellular response to interferon‐gamma^a^	
TRINITY_DN36688_c0_g1_i1	MORF/ORRM1/DAG‐like MORF domain‐containing protein (*E* = 4.3)	Involved in organellar RNA editing in mitochondria^b^	
TRINITY_DN4363_c0_g3_i3	Cytochrome c oxidase subunit 1 (*E* = 2e‐32) [Culex]^b^, ^d^	Mitochondrial electron transport chain; drives oxidative phosphorylation^b^	Changes in COX in bar‐headed geese flying at high altitudes (Scott et al. 2011)
TRINITY_DN50086_c0_g1_i1	1. Growth Factor Receptor Bound protein 7 (GRB7) (*E* = 0.045)^a^ 2. Histone‐lysine N‐methyltransferase SETD1B (*E* = 0.13)^a^	1. Integrin signaling pathway; cell migration; cell proliferation; Immune response IL‐23 signaling pathway; signaling by Rho GTPases^a^ 2. Plays an essential role in regulating transcriptional programming of multipotent hematopoietic progenitor cells and lymphoid lineage specification during hematopoiesis^a^	1. Interacts with NRG1 (thermoregulation & inflammation) (Battista 2022). GRB2 differentially expressed in malaria infection (Paxton et al. 2023); Transforming growth factor β receptor III differentiated low/high elevations and under selection in low‐elevation populations (Cassin‐Sackett, Callicrate, and Fleischer 2019) 2. SETD1B differentially expressed in fatalities relative to controls (Paxton et al. 2023)
TRINITY_DN57110_c0_g1_i1	Acyl‐CoA‐binding protein (*E* = 2e‐4)^c^	Plays role in acyl‐CoA dependent lipid metabolism; similar protein ACBD5 may play a role in the differentiation of megakaryocytes and formation of platelets^c^	Expression differences in other coagulation factors in yaks in different temperatures (Gao et al. 2023)
TRINITY_DN57959_c0_g1_i1	Chitin synthase regulator 2 (*E* = 4.6)	Septum formation & cell division in fungi	
TRINITY_DN58887_c0_g1_i1	1. Caytaxin (*E* = 0.013) 2. Mitochondrial import inner membrane translocase subunit TIM54 (*E* = 0.024)	1. Development of neural tissues 2. TIM54 helps mediate the import and insertion of multi‐pass transmembrane proteins into the mitochondrial inner membrane	2. TIMM44 enriched/common missense mutation in Tibetan human populations ‐ inferred under selection (Zheng et al. 2023)
TRINITY_DN6320_c0_g1_i1	RNase H type‐1 domain‐containing protein (*E* = 9e‐40); Envelope glycoprotein (*E* = 4e‐38)	Membrane protein; endogenous retrovirus	Chromosomal inversion in Tibetan sheep enriched in genes related to angiogenesis and UV‐mediated immune response, including 3 RNases (1, 4, and 7; Liang et al. 2024)
TRINITY_DN8097_c0_g1_i1	von Willebrand factor (*E* = 0.043)^c^	Encodes a glycoprotein involved in hemostasis; promotes adhesion of platelets to the sites of vascular injury; transport of proteins in the blood^c^	von Willebrand factor domain containing 8 differentially expressed in experimentally infected fatalities (Paxton et al. 2023); expression differences in other coagulation factors in yaks in different temperatures (Gao et al. 2023)
TRINITY_DN8772_c2_g1_i1	NADH–ubiquinone oxidoreductase chain 5 (*E* = 4e‐41)^b^	Mitochondrial electron transport chain^b^	

*Note:* E = expect value from Blast search, with lower values indicating more significant matches.

^a^ Immune function.

^b^ Cellular respiration.

^c^ Coagulation and platelet function.

^d^ Blast match to *Culex* mosquito gene.

## 4 Discussion

Avian malaria has elicited rapid changes in the Hawai'i 'amakihi's genome (Cassin‐Sackett, Callicrate, and Fleischer 2019) and transcriptome (Paxton et al. 2023) and is associated with changes in microbiome (Navine et al. 2023). Yet we lack information on how gene expression varies in natural populations of 'amakihi with different intensities of exposure to *Plasmodium relictum*. Here, we report differential expression of a suite of diverse genes in infected birds and those with no detected infection and a nonsignificant trend of differential gene expression across elevation in transcripts involved in cellular respiration, coagulation, and immune response. Many of these genes have been implicated previously in other systems, suggesting parallel evolution in response to both pathogens and factors that vary with elevation.

In infected birds, expression of multiple genes related to inflammation and the immune response was higher than in uninfected individuals (Table 1); this finding agrees with pathology studies of infected 'amakihi (Atkinson et al. 2000) and supports the hypothesis of a systemic response to pathogenic infection. Of the top 13 most differentially expressed transcripts, all of which were upregulated in infected birds, eight of them blasted to genes previously implicated in response to malaria in Hawai'i 'amakihi (Cassin‐Sackett, Callicrate, and Fleischer 2019; Paxton et al. 2023; Atkinson et al. in review), and three others have been identified in mammalian infections with other apicomplexan parasites (including *Plasmodium berghei*; Desruisseaux et al. 2010; Laroque 2016; Mackinnon et al. 2016; Seixas et al. 2012). These genes had functions in binding and cell adhesion (*BOD1*, *PCDHGA6*), including binding as part of the immune response pathway (*CNTN2*, *UMOD*); cellular upkeep and apoptosis (*RBMS3*, *SMG1*, *FANCD2*); cell signaling (*ADGRF5*, *ZNF451*); and general immune response (*RAB20*, *FANCD2*, *PNPH*).

Upregulation of immune signaling and binding/cell adhesion genes in infected 'amakihi may indicate pathogen recognition and the initiation of an appropriate immune response (Paxton et al. 2023). Several genes involved in cell adhesion were upregulated in infected birds, including *CNTN2* and *BOD1*. *CNTN2* interacts directly with a gene (*NCAM1*) that is crucial for immune surveillance and that induces expansion of T lymphocytes and B lymphocytes. Other contactin genes have previously been implicated in host response to malaria in both mice (Desruisseaux et al. 2010) and humans (Damena et al. 2021) and yet others were inferred to be under selection in Hawai'i 'amakihi (Cassin‐Sackett, Callicrate, and Fleischer 2019). *BOD1* and sumo proteins interact with a gene (*TRIM28*) that suppresses regulatory T cells, and *BOD1* was downregulated in experimentally infected 'amakihi (Paxton et al. 2023). Therefore, higher expression of these genes in infected 'amakihi could suggest they are mounting an effective immune response (although without repeated captures and estimates of infection intensity over time, it is difficult to confirm the success of the immune response).

In addition, two calcium‐related binding and adhesion genes were upregulated in infected birds: *PCDHGA6*, a calcium‐dependent cell adhesion protein, and *UMOD*, a calcium ion binding and immunoglobulin G binding protein. Calcium is required for *Plasmodium* growth and invasion (Scheibel et al. 1987; de Oliveira et al. 2021); therefore, alteration of available calcium by the host could be a mechanism to reduce parasite survival. Indeed, the gene most significantly differentially expressed in 'amakihi that survived or succumbed to experimental malaria infection was a gene that controlled the release of intracellular calcium (Paxton et al. 2023), and calcium signaling and transport genes were highly differentiated between low‐ and high‐elevation 'amakihi (Cassin‐Sackett, Callicrate, and Fleischer 2019). *UMOD* itself was differentially expressed in 'amakihi experimentally infected with malaria via the bite of an infectious mosquito versus subinoculation of infected blood (Paxton et al. 2023), but not in other comparisons. *PCDHGA6* was upregulated in mice infected with *Plasmodium berghei* (Desruisseaux et al. 2010), and a gene that activates *PCDHGA6* and *PCDH10* was downregulated in experimentally infected 'amakihi that succumbed to infection (Paxton et al. 2023). Other cadherins have been identified as candidate resistance genes to malaria in humans (Mackinnon et al. 2016) and in other pathogens in wildlife (Cassin‐Sackett et al. 2025). Thus, the upregulation of these genes in wild infected 'amakihi could indicate a successful immune reaction.

Effective cell signaling is essential for a successful immune response, and 'amakihi that do not survive malaria infection have shown lower expression of several immune‐related signaling genes (Paxton et al. 2023). Here, conversely, infected 'amakihi were characterized by higher expression of signaling genes *ADGRF5* and E3 SUMO protein ligase *ZNF451*. *ADGRF5* is involved in the cell surface receptor signaling pathway, and mice without functional *ADGRF5* display heightened inflammation and upregulation of genes involved in the immune response (Kubo et al. 2019). *ZNF451* exerts negative regulation of the transforming growth factor beta receptor signaling pathway; this gene was the most downregulated gene by *Toxoplasma gondii*‐infected cells 48 h after infection (Elsheikha et al. 2019). Similarly, another SUMO ligase was differentially expressed in infected 'amakihi at different time points (Paxton et al. 2023).

Successfully combating pathogens requires a host to mount an immune response while reducing the negative effects of inflammation, and an excessive inflammatory response has been linked to host mortality in diseases such as white‐nose syndrome in bats (Hoyt et al. 2021), chytridiomycosis in amphibians (Savage et al. 2020), and COVID‐19 in humans (Huang et al. 2020). This phenomenon was similarly observed in Hawai'i 'amakihi experimentally infected with *Plasmodium* (Paxton et al. 2023). In the present study, naturally infected birds did not display excessive upregulation of inflammatory genes, but instead were characterized by higher expression of genes regulating T‐cell immunity, B‐cell immunity, and antibody responses (*PNPH*, *FANCD2*, and *RAB20*). *FANCD2* is a gene involved in DNA repair and the switching of B‐cell immunoglobulin isotypes (Yamamoto et al. 2005); it can reduce viral replication in a host (Spriggs and Laimins 2017) and is upregulated in tissues of sheep infected with a platyhelminth parasite (Alvarez Rojas et al. 2015). The blast match to this gene (and several other genes in Tables 1 and 2) was characterized by only moderate statistical support, and confidence in annotations is currently a limitation in this and other studies of nonmodel organisms. Nonetheless, similarities with other studies can support these inferences. For instance, a functionally related gene, Fanconi anemia complementation group E (*FANCE*), was highly differentiated across elevation in 'amakihi (Cassin‐Sackett, Callicrate, and Fleischer 2019), and *FANCL* and *FANCI* were upregulated in 'amakihi survivors reducing parasitemia and in fatalities relative to uninfected 'amakihi, respectively (Paxton et al. 2023). *PNPH* regulates T‐cell and B‐cell immunity and antibody responses and can influence resistance to infection (Tecle et al. 2021). This gene was upregulated in some strains of *Plasmodium*‐infected mice (De‐Oliveira et al. 2006) but expression was reduced in other strains (Carvalho et al. 2009). *FANC* genes and *PNPH* may therefore be more nuanced in their response to infection and the direction of their effects on survival. Finally, *RAB20* is a membrane‐trafficking protein needed for cytokine production and the maturation of phagosomes that engulf pathogens. In an experimental mouse model, *RAB20* was correlated with cytokine expression and upregulated during acute inflammation (Liang et al. 2012). Moreover, *RAB20* appears to respond to *Plasmodium* itself: infection with *P. berghei* resulted in upregulation of host *RAB20* and other Rab genes (Seixas et al. 2012), and surviving 'amakihi upregulated *RAB20* during infection (Paxton et al. 2023). At the sequence level, polymorphisms in *RAB20* were strongly associated with survivorship phenotype in experimentally infected 'amakihi (Atkinson et al. in review). Therefore, increasing expression of *RAB20*, as observed here in wild infected 'amakihi, could improve the probability of surviving infection with *P. relictum* .

In the genes differentially expressed between infected and uninfected birds, we observed enrichment of several gene ontology categories in genes upregulated in uninfected birds. These genes were related to positive regulation of cell death and negative regulation of transcription and biosynthetic and metabolic processes, suggesting that uninfected birds are interrupting the cell cycle and causing cell death. It is possible that uninfected birds are clearing a previous infection or reducing parasitemia to undetectable levels by killing infected cells, whereas infected birds are regulating genes that minimize the effects of existing infections.

In our elevational comparison, a small number of genes showed a trend, although not significant, of differences in expression. In high‐elevation birds, the expression of proteins involved in cellular respiration (*COX1*, *COX2*, *NADH5*) was higher. Changes in gene expression, enzyme kinetics, and coding sequences of *COX* genes have been observed previously in the high‐flying bar‐headed goose (Scott et al. 2011), suggesting that birds at higher elevations invoke these genes to cope with hypoxia. Indeed, *COX* and *NADH* genes play a role in adaptation to high elevations across endothermic vertebrates (Cheviron and Brumfield 2009, 2012). Other mitochondrial genes also appear to be involved in coping with different elevational environments. For instance, one 'amakihi transcript blasted to mitochondrial import inner membrane translocase subunit *TIM54*; a similar gene (*TIMM44*) contained a missense mutation at high frequencies in Tibetan human populations living at high elevations (Zheng et al. 2023). In addition, one transcript blasted to a RNase H type‐1 domain‐containing protein. In high‐elevation Tibetan sheep, genomes contained a chromosomal inversion enriched in genes related to angiogenesis and UV‐mediated immune response, including 3 RNases (1, 4, and 7; Liang et al. 2024).

Three transcripts that were upregulated (albeit not significantly) at high elevations had inferred roles in pathogen response (Ig‐like domain‐containing protein, Ras‐related protein *Rab‐12*, and Growth Factor Receptor Bound protein 7 (*GRB7*)). Immunoglobulin domains have been implicated previously in this system: *SEMA3A* (immunoglobulin domain, semaphorin 3A) was the gene most highly differentiated in sequence between multiple pairs of low‐ and high‐elevation 'amakihi populations (Cassin‐Sackett, Callicrate, and Fleischer 2019). Moreover, *SEMA4B* was upregulated in infected 'amakihi relative to control birds (Paxton et al. 2023). In addition to modulating immune response, semaphorins may be involved in temperature regulation, as expression of Ig‐like domain‐containing protein was lower in yaks exposed to heat stress (Gu 2024). *GRB7*, part of the immune response interleukin 23 signaling pathway, interacts with other genes to influence thermoregulation and inflammation (Battista 2022). Similar genes have been previously implicated in malaria response in Hawai'i 'amakihi: several *GRB2* binding proteins were differentially expressed at different points throughout the course of infection (Paxton et al. 2023), and Transforming growth factor β receptor III showed sequence divergence and other signals of selection between low‐ and high‐elevation 'amakihi (Cassin‐Sackett, Callicrate, and Fleischer 2019). Another blast match to this same transcript, Histone‐lysine N‐methyltransferase (*SETD1B*), was differentially expressed in 'amakihi succumbing to experimentally infected malaria, relative to control birds (Paxton et al. 2023). Finally, *RAB12* acts upstream of or within the cellular response to interferon‐gamma but, to our knowledge, has not been implicated previously in response to avian malaria or elevation.

Two transcripts that were slightly upregulated in high‐elevation birds blasted to genes related to platelet formation (Acyl‐CoA‐binding protein) and platelet adhesion at injury sites as well as protein transport in the blood (von Willebrand factor). Other coagulation factors have exhibited differences in expression in yaks exposed to different temperatures (Gao et al. 2023). Interestingly, a von Willebrand factor was upregulated in 'amakihi experimentally infected with malaria that died from infection (Paxton et al. 2023), hinting at potential antagonistic pleiotropy (Hancock et al. 2011) between adaptation to high elevations and susceptibility to infection (Roulin et al. 2011; Seddon and Hews 2017). Hypoxia and changes in temperature can influence susceptibility to infections (Cohen et al. 2017; Dzhalilova and Makarova 2020; Mourtzoukou and Falagas 2007; Paull et al. 2012), and it is possible that birds adapted to high‐elevation conditions such as lower oxygen pressure and temperature are upregulating genes in a manner that results in heightened susceptibility to malaria. Additional work could help to disentangle the effects of high‐elevation adaptation from the lack of adaptation to *Plasmodium*; for instance, low‐elevation birds that have evolved immunity to the parasite could be placed in hypoxic temperature‐controlled chambers prior to experimental infection with *P. relictum* .

Our results demonstrate the upregulation in infected birds of several genes involved in the immune response. Without recapture data, we are unable to confirm whether these infected birds survived or succumbed to infection; hence, it is unclear whether higher expression of these genes is linked to the survival outcome. However, prior results in this system suggest that these infected 'amakihi already are survivors: Birds are rarely captured during the acute infection phase (Samuel et al. 2015), likely due to behavioral changes associated with early‐stage infection (Yorinks and Atkinson 2000). In addition, the prevalence of malaria in 'amakihi at Bryson's cinder cone during the sampling period was very high (*N* = 27, prevalence = 81% (McClure et al. 2020)). Taken together, this evidence suggests that most of the infected birds in our study—all but one of which were second year or older—were likely chronically infected survivors (Atkinson et al. 2001). Several of the genes identified here were also differentially expressed in experimentally infected 'amakihi (Paxton et al. 2023), allowing for additional inference of the effects of upregulating expression. For instance, among the genes upregulated in infected wild‐caught birds reported here, *RAB20* was also upregulated in 'amakihi surviving experimental infection, and three upregulated genes (*COG5*, *BOD1*, and *PCDH10* which activates *PCDHGA6*) were downregulated in 'amakihi that perished (Paxton et al. 2023). Thus, evidence from the expression of these genes could suggest that the wild‐caught 'amakihi here may be likely to survive infection. This inference points to the conclusion that the genes displaying higher expression in infected birds in this study likely represent genes regulated by the host to manage chronic infections after parasitemia has been reduced relative to the acute phase of infection. Experimentally infected 'amakihi that later died (Paxton et al. 2023) rapidly upregulated inflammation genes and sustained high levels of expression until death, potentially indicating a lack of ability to fine‐tune the immune response (this pattern has also been observed in hosts of other infectious diseases, e.g., Savage et al. 2020). In our elevational comparison, we observe higher base levels of gene expression of several inflammation genes in high‐elevation birds. Excessive inflammation can result in pathology (Penha‐Gonçalves 2019) that could explain the increased susceptibility observed in high‐elevation 'amakihi populations.

Outstanding questions remain about the mechanistic links between variables such as infection intensity, stage of infection, and elevation to the expression of specific genes in surviving and perishing birds in wild populations (Westerdahl et al. 2012). For instance, native avian species in Hawai'i are characterized by higher parasitemia than native species (van Riper III et al. 1986; McClure et al. 2020; Seidl et al. 2024), but it is unclear whether individuals from native species that survive infection upregulate the same genes as surviving individuals from non‐native species, or whether individuals with high‐intensity infections upregulate the same genes as those with low‐intensity infections. In addition, experimentally challenged birds upregulated different genes during different periods of infection (Paxton et al. 2023), but information about gene expression during different stages of infection remains elusive in natural populations. At low elevations in Hawaii, mosquitoes are present year‐round, so birds can become infected at any time, making it difficult to discern when an individual became infected. For instance, analysis of nearly 2000 'amakihi from low elevations revealed equal infection probability between juvenile and adult 'amakihi and a lack of seasonality in infection rates (Samuel et al. 2015). In some systems, infection intensity can be a proxy for time since infection, but in malaria, parasitemia levels are low both at early and late stages of infection, so time is not an adequate proxy (Erokhina et al. 2025). Longer‐term capture‐mark‐recapture studies in this system might clarify nuances in the influence of infection stage on gene expression. Additionally, the small sample size of uninfected birds at low elevations was a limitation of this study. Further sampling at both low and high elevations could allow for an explicit comparison of differential expression between infected and uninfected birds within elevations, avoiding the potential confounding effects of elevation on gene expression in uninfected birds.

The relationship between evolutionary adaptation, infection prevalence, and intensity of infection is complex. Adaptation to introduced malaria enables 'amakihi to survive with infection, which can in turn increase malaria prevalence in communities with 'amakihi (McClure et al. 2020), potentially leading to a positive feedback loop of increased selection for immunity via increased exposure of native birds to *Plasmodium*. However, although this adaptation does lead to reduced parasitemia in surviving relative to perishing 'amakihi (Atkinson et al. 2013; Paxton et al. 2023), 'amakihi are characterized by higher parasitemia levels than nearly all introduced species in Hawai'i (Seidl et al. 2024). Moreover, birds can be highly infectious to mosquitoes even at very low parasitemia levels; therefore, in the absence of an adaptation that leads to complete clearance of the parasite (or eradication of the mosquito, Kyriazis et al. 2025), avian malaria is likely to persist in avian communities in Hawai'i (Seidl 2023).

Collectively, our results reveal differential expression between infected and uninfected 'amakihi at loci influencing the inflammatory and immune responses, many of which have been implicated previously in this system (Cassin‐Sackett, Callicrate, and Fleischer 2019; Paxton et al. 2023) or in infections with other apicomplexan parasites (Damena et al. 2021; Kubo et al. 2019; Marsilia et al. 2023; Sutanto et al. 2023), suggesting the central role of these genes in combating parasitic infection. In addition, we uncovered a nonsignificant trend of differential expression between high and low elevations in several immune genes that mirror other studies in the system (Cassin‐Sackett, Callicrate, and Fleischer 2019; Paxton et al. 2023), in coagulation factors similar to those in systems with populations in different temperatures (Gao et al. 2023), and in mitochondrial genes that mimic other systems with high‐elevation populations (Liang et al. 2024; Scott et al. 2011; Zheng et al. 2023). The number of genes recovered here that have also been identified in other studies suggests a high degree of parallel evolution (Rivas et al. 2018) in response to both pathogens and factors that covary with elevation. This may be in part due to the low mean number of reads per sample, which enabled the detection of only the strongest signals of differential expression, or it could be because there is more parallelism in gene expression (Levis and Pfennig 2020) than in sequence evolution (Cooper et al. 2003) in this system. Detecting large effects can be useful for conservation measures, as interventions are likely to target loci of large effect. Nonetheless, future work that aims to sequence at higher depth might identify gene complexes and pathways that covary with elevation and malaria infection, as our low number of reads likely could not detect biologically meaningful transcripts with low abundance and/or small magnitudes of differential expression. Future efforts could also attempt to determine whether antagonistic pleiotropy predisposes high‐elevation 'amakihi to be adapted to montane climatic conditions at a cost of heightened susceptibility to malaria.

## Author Contributions

**Loren Cassin‐Sackett:** conceptualization (equal), formal analysis (lead), funding acquisition (equal), investigation (lead), visualization (lead), writing – original draft (lead), writing – review and editing (lead). **Katherine M. McClure:** formal analysis (supporting), investigation (supporting), writing – review and editing (supporting). **Taylor E. Callicrate:** investigation (supporting), writing – review and editing (supporting). **Eben H. Paxton:** funding acquisition (supporting), investigation (supporting), writing – review and editing (supporting). **Robert C. Fleischer:** conceptualization (equal), funding acquisition (equal), investigation (supporting), writing – review and editing (supporting).

## Conflicts of Interest

The authors declare no conflicts of interest.

## Data Availability

A detailed description of the pipeline, along with the scripts, is available on GitHub (https://github.com/CassinSackett/RNA_seq), and the pipeline is published on FigShare (DOI https://doi.org/10.6084/m9.figshare.28765724). All sequence data have been deposited in NCBI's Sequence Read Archive under Project number PRJNA1248578.

## Appendix A Samples, Infection Status, Sampling Elevation, and Sequencing Statistics for Samples Used in This Study

**Table:**

sample	# Raw reads	Paired‐reads post‐Trimmomatic	pct‐retained	PEalign‐overall‐alignment%	Group in infection status DE analysis	Group in elevation DE analysis
r5_KOAR‐1_1291‐16732_high	10,757,925	3,327,651	0.3093209	94.15	Undetected	High
r1_PUAK_2020‐40771_high	4,247,092	2,175,066	0.51213065	95.94	Undetected	High
r33_PEDR_2591‐07630_high	1,614,662	1,070,717	0.66312145	75.65	Undetected	High
r10_PUAK_2020‐40667_high	2,704,697	1,140,641	0.42172598	96.25	Undetected	High
r34_PEDR_2591‐07628_high	1,396,132	898,391	0.64348572	93.31	Undetected	High
r2_PUAK_2591‐07641_high	842,342	589,259	0.6995484	97.18	Undetected	High
r6_PEDR_2591‐07626_high	810,808	380,812	0.46966976	93.58	Undetected	High
r8_PUAK_2591‐07618_high	926,909	335,325	0.3617669	82.18	Undetected	High
r31_KOAR_2591‐07631_high	742,741	400,865	0.53971034	93.42	Undetected	High
r26_PUAK_2591‐07637_high	915,884	220,317	0.24055121	91.05	Undetected	High
r9_KOAR_2591‐07632_high	756,309	586,830	0.77591302	70.37	Undetected	High
r7_KOAR_2591‐07633_high	454,918	141,840	0.31179245	79.91	Undetected	High
r3_PUAK_2591‐07639_high	131,264	34,838	0.26540407	95.76	Undetected	High
r4_PUAK_2591‐07640_high	81,975	30,835	0.37615127	50.41	Undetected	High
r43_PEDR_1851‐54924_high	156,037	24,552	0.1573473	40.29	Undetected	High
r35_BRY_2511‐57223_low	139,475	125,298	0.89835454	65.93	Undetected	Low
r17_BRY_2511‐57202_low	419,831	157,320	0.37472221	97.53	Undetected	Low
r16_BRY_2511‐57201_low	984,823	179,526	0.18229266	86.28	Undetected	Low
r28_BRY_2511‐57208_low	1,147,621	478,235	0.41671859	89.32	Undetected	Low
r13_BRY_2120‐99697_low	847,981	394,361	0.46505877	93.94	Undetected	Low
r22_BRY_2511‐57205_low	3,783,838	2,225,554	0.5881737	96.36	Undetected	Low
r15_BRY_2120‐99696_low	4,870,482	2,604,444	0.5347405	95.44	Undetected	Low
r37_BRY_2511‐57207_low	3,009,705	1,965,321	0.65299456	96.16	Undetected	Low
r21_BRY_2511‐57204_low	1,635,914	1,077,601	0.65871494	95.58	Undetected	Low
r38_BRY_2511‐57206_low	1,215,375	912,199	0.75054942	91.36	Undetected	Low
r24_BRY_2511‐57221_plas_low	11,907,200	7,579,795	0.63657241	94.63	Infected	Low
r29_BRY_2511‐57214_plas_low	8,791,261	5,179,364	0.58914916	95.65	Infected	Low
r20_BRY_2511‐57222_plas_low	1,584,741	1,083,115	0.683465	98.05	Infected	Low
r25_BRY_2511‐57226_plas_low	2,301,653	1,877,849	0.81586972	97.63	Infected	Low
r19_BRY_2511‐57219_plas_pox_low	1,638,611	1,082,017	0.66032573	91.97	Infected	Low
r18_BRY_2511‐57216_plas_low	1,777,718	705,278	0.39673222	88.01	Infected	Low
r39_BRY_2511‐57213_plas_low	641,173	516,459	0.80549087	90.58	Infected	Low
r12_BRY_2511‐57218_plas_low	561,493	250,756	0.44658794	74.53	Infected	Low
r41_BRY_2511‐57211_plas_low	257,315	178,201	0.69254027	97.61	Infected	Low
r27_BRY_2511‐57225_plas_low	169,402	134,243	0.79245227	73.4	Infected	Low
r30_BRY_2511‐57210_plas_low	339,939	53,695	0.15795481	93.15	Infected	Low
r40_BRY_2511‐57212_plas_low	218,465	30,287	0.13863548	51.52	Infected	Low
r36_BRY_2511‐57217_plas_low	102,994	17,067	0.16570868	82.28	Infected	Low
r23_BRY_2511‐57209_plas_low	104,156	8529	0.08188679	79.91	Infected	Low
